# *In vitro* anti-MRSA activity of carvone with gentamicin

**DOI:** 10.3892/etm.2014.1498

**Published:** 2014-01-23

**Authors:** SU-HYUN MUN, OK-HWA KANG, DAE-KI JOUNG, SUNG-BAE KIM, JANG-GI CHOI, DONG-WON SHIN, DONG-YEUL KWON

**Affiliations:** 1 Department of Oriental Pharmacy, College of Pharmacy, Wonkwang Oriental Medicines Research Institute, Wonkwang University, Iksan, Jeonbuk 570-749, Republic of Korea; 2 BK21 Plus Team, Professional Graduate School of Oriental Medicine, Wonkwang University, Iksan, Jeonbuk 570-749, Republic of Korea; 3 BK21 Plus Program & Department of Smart Life-Care Convergence, Gradulate School, Wonkwang University, Iksan, Jeonbuk 570-749, Republic of Korea; 4 Department of Oriental Medicine Resources, College of Bio Industry Science, Sunchon National University, Sunchon, Jeonnam 540-742, Republic of Korea

**Keywords:** carvone, methicillin-resistant *Staphylococcus aureus*, gentamicin, synergy

## Abstract

Carvone is one of the naturally occurring monoterpenes, the largest class of secondary metabolites in plants, and exists in two enantiomers, R-carvone (R-car) and S-car. The objective of this study was to investigate the antimicrobial activity of R-car and S-car with gentamicin (GET) against methicillin-resistant *Staphylococcus aureus* (MRSA). MRSA is a major human pathogen that causes serious problems, including hospital-acquired pneumonia, abscesses and surgical wound infections. Nosocomial MRSA infections often exhibit multidrug resistance. In the present study, antimicrobial susceptibility testing was performed with R-car, S-car and GET using the broth microdilution method. Minimal inhibitory concentration values for R- and S-car against six different strains of *S. aureus* ranged between 500 and 1,000 μg/ml. Anti-MRSA activity was evaluated using the checkerboard and time-kill assays to investigate the potential synergistic effects of different combinations of the carvone enantiomers and GET. R-car plus S-car, R-car plus GET and S-car plus GET exhibited significant synergistic activity against MRSA. These findings suggest that the single-agent anti-MRSA activities of R-car, S-car and GET are effectively increased through combination therapy. This study showed that carvone may be a potential adjuvant antimicrobial agent.

## Introduction

**Figure 1:**
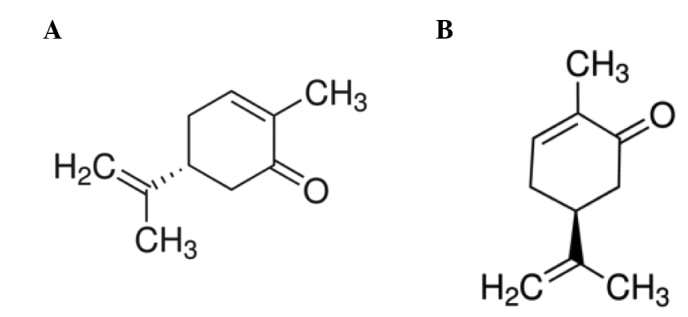
(A) R-(−)-carvone; (B) S-(+)-carvone.

The monoterpene carvone is an enantiomeric compound. R-carvone (R-car) smells like spearmint and is found naturally in numerous essential oils, while S-car is the principal constituent of caraway seed oil (Fig. 1) (1). Carvone is a chiral molecule and its enantiomers are non-superimposable mirror images of each other that exhibit distinct chemical properties. Enantiomers are important in pharmacology as chimeric drugs may comprise one enantiomer that is responsible for the desired physiological change and a second enantiomer that is inactive or elicits adverse effects (2,3). Previous studies on the carvone enantiomers have demonstrated an enantioselective penetration-enhancing effect and an enantioselective influence on the structure and function of a microbial river water system (4,5). Furthermore, it has been revealed that stereoselectivity in phase-I and -II metabolism has significant effects on the pharmacokinetics of R- and S-car (6). However, to the best of our knowledge, the antimicrobial activity of carvone against methicillin-resistant *Staphylococcus aureus* (MRSA) has not been investigated.

Methicillin was discovered by Alexander Fleming in 1928, and has been used to clinically treat staphylococcal infections since 1959 (7). Methicillin resistance is generated by the acquisition of genes encoding penicillin-binding proteins (PBPs), which have low affinities for β-lactam antibiotics. Therefore, MRSA is generated when methicillin-susceptible *S. aureus* acquires the methicillin resistance gene *mec*A (8). *S. aureus* is a gram-positive pathogen that causes a variety of systemic infections, including hospital-acquired pneumonia, surgical wound infections and exotoxin syndromes (9). In recent years, the virulence of MRSA has acounted for nearly 70% of *S. aureus* infections, and the rapid emergence of antibiotic resistant strains has made it difficult to treat these infections (10). Therefore, novel therapeutic approaches are necessary to minimize bacterial resistance against conventional antibiotics. The aim of the present study was to determine the anti-MRSA activities of the combination of R-car and S-car, and the combination of either carvone enantiomer with gentamicin (GET).

## Materials and methods

### Reagents

R- and S-car were obtained from Tokyo Chemical Industry Co., Ltd. (Tokyo, Japan). Mueller-Hinton agar (MHA) and Mueller-Hinton broth (MHB) were purchased from Becton, Dickinson and Company (Franklin Lakes, NJ, USA). Tris(hydroxymethyl)aminomethane was obtained from Amresco LLC (San Francisco, CA, USA), and sodium azide (NaN_3_) and peptidoglycan were purchased from Fluka Chemie GmbH (Buchs, Switzerland). GET, Triton X-100, N,N-dicyclohexylcarbodiimide and purified lipopolysaccharide were obtained from Sigma-Aldrich Co. LLC (St. Louis, MO, USA).

### Bacterial strains and growth conditions

Among the six strains of *S. aureus* that were used in this study, four were clinical MRSA isolates obtained from four patients who were treated at Wonkwang University Hospital (Iksan, Korea). These strains were referred to as staphylococcal strains from the Department of Plastic Surgery (DPS)-1, -2, -3 and -4. The remaining two *S. aureus* strains, ATCC 33591 (MRSA) and the methicillin-susceptible strain ATCC 25923, were commercially available (American Type Culture Collection, Manassas, VA, USA). All bacteria were stored in 30% glycerol and frozen at −70°C. Prior to each experiment, the bacterial strains were suspended in MHB and incubated at 37°C for 24 h. MHA was used in the agar diffusion method for determining the minimal inhibitory concentration (MIC).

### Antimicrobial susceptibility

MICs were determined using the broth microdilution method, as described by the Clinical and Laboratory Standards Institute (11). Serial two-fold dilutions of carvone in MHB were prepared using sterile 96-well microplates and microtubes. The MRSA inocula were adjusted to the 0.5 McFarland standard [~1.5×10^8^ colony-forming units (CFU)/ml] in MHB. The final inocula were adjusted to 1.5×10^6^ CFU/spot. The MIC was defined as the lowest concentration of carvone that permits microorganism growth subsequent to incubation at 37°C for 24 h.

### Synergy

The antimicrobial activities of R-car, S-car and GET were investigated using the checkerboard dilution method to determine the interactions between these agents (12,13). Serial dilutions of two selected agents were mixed in cation-supplemented MHB. The inocula were prepared from colonies that had been grown overnight on MHA. The final bacterial concentration following inoculation was 1.5×10^6^ CFU/spot. The *in vitro* interaction between the drugs was quantified by determining the fractional inhibitory concentration (FIC). The FIC index (FICI) was calculated with the following formula: FICI = FIC_A_ + FIC_B_ = [A]/MIC_A_ + [B]/MIC_B_, where [A] and [B] are the concentrations of drug A and B, respectively, and MIC_A_/FIC_A_ and MIC_B_/FIC_B_ are the MIC/FIC of drug A and B, respectively. The FICI was interpreted as follows: ≤0.5, synergy; >0.5–0.75, partial synergy; >0.75–1, additive effect; >1–4, no effect; and >4, antagonism (14). The different values of synergy between each pair of agents were calculated. Each experiment was performed in triplicate.

### Time-kill assay

The synergy between each pair of antimicrobial agents was determined using time-kill curves of bacterial growth in 96-well plates at five different time-points (0, 4, 8, 16 and 24 h) (12). Bacterial cultures were diluted with fresh MHB to ~1.5×10^6^ CFU/ml, and incubated at 37°C for 24 h. Aliquots (0.1 ml) of the culture were taken following 0, 4, 8, 16 and 24 h of incubation, and serial 10-fold dilutions were prepared in saline. For samples obtained from each time-point, the number of viable cells was determined on a drug-free MHA plate following incubation for 24 h. Colony counts were performed on plates and 30–300 colonies were counted. The lower limit of sensitivity for the colony counts was 100 CFU/ml. Antimicrobial agents were considered to be bactericidal at the lowest concentration that reduced the original inoculum by 3 log10 CFU/ml (99.9%) for each of the indicated time-points. Antimicrobial agents were classified as bacteriostatic if the inoculum was reduced by only 0–3 log10 CFU/ml. To confirm the results, time-kill assays for each experiment were performed in triplicate. Data are presented as the mean ± standard deviation.

### Transmission electron microscopy (TEM)

MRSA exponential phase cultures were prepared by diluting overnight cultures with MHB and incubating at 37°C until the mid-logarithmic growth phase was reached. The MHB-grown exponential-phase MRSA cultures were treated with R-car at 1/2 MIC and 1 MIC for 30 min. Subsequently, 2 ml culture medium was collected by centrifugation at 10,000 × g for 10 min. Following removal of the supernatant, pellets were fixed with a modified Karnovsky’s fixative. The specimens were examined with an energy-filtering transmission electron microscope (Libra 120; Carl Zeiss, Oberkochen, Germany) operated at an accelerating voltage of 120 kV. The transmitted electronic signals were recorded with a 4k × 4k slow-scan charge-coupled device camera (Ultrascan 4000 SP; Gatan, Inc., Pleasanton, CA, USA), which was attached to the electron microscope.

## Results

### Antimicrobial susceptibility testing

Antimicrobial susceptibility tests of six strains of *S. aureus* against R-car, S-car and GET were performed using the standard broth microdilution method. The MICs for R-car, S-car and GET against the six *S. aureus* strains are presented in Table I. The growth of *S. aureus* was inhibited by R- and S-car at concentrations ranging between 500 and 1,000 μg/ml.

### Combined effect of R-car, S-car and GET

The synergistic effects of the combination therapies are shown in Tables II–IV. The combination of two antimicrobial agents (R-car plus S-car, R-car plus GET and S-car plus GET) markedly reduced the MIC against all *S. aureus* strains. The combination of R- and S-car exhibited a synergistic effect with an FICI of 0.12–0.37 (Table II). When R-car was combined with GET, the mean FICI was 0.09–0.38 (Table III). Similarly, the combination of S-car and GET had a synergistic effect with a mean FICI of 0.18–0.31 (Table IV).

### Time-kill assay

**Figure 2:**
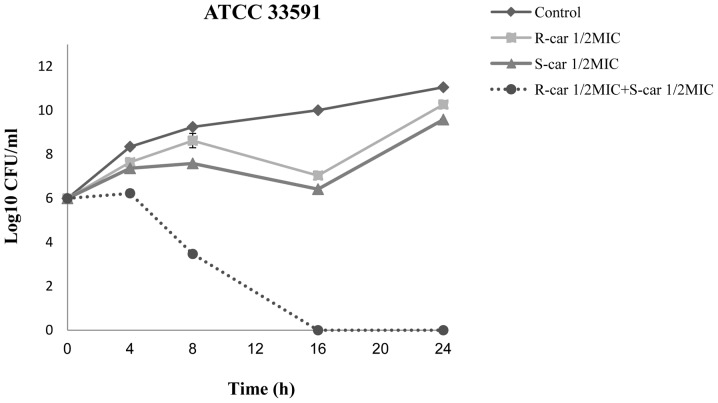
Time-kill curves for methicillin-resistant *Staphylococcus aureus* (strain ATCC 33591) with R-car and S-car. MIC, minimal inhibitory concentration; CFU, colony-forming units; R-car, R-carvone; S-car, S-carvone.

**Figure 3:**
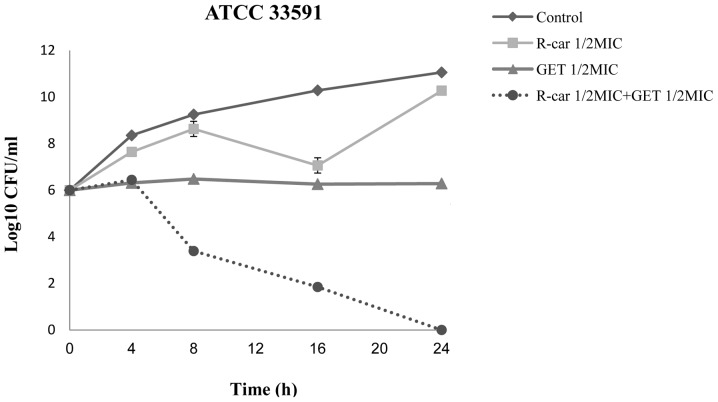
Time-kill curves for methicillin-resistant *Staphylococcus aureus* (strain ATCC 33591) with R-car and GET. MIC, minimal inhibitory concentration; CFU, colony-forming units; R-car, R-carvone; GET, gentamicin.

**Figure 4:**
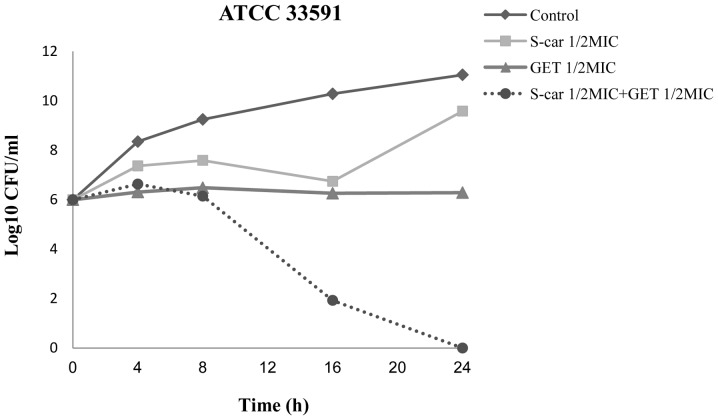
Time-kill curves for methicillin-resistant *Staphylococcus aureus* (strain ATCC 33591) with S-car and GET. MIC, minimal inhibitory concentration; CFU, colony-forming units; S-car, S-carvone; GET, gentamicin.

The synergistic effects of R-car, S-car and GET against the *S. aureus* strain ATCC 33591 were further evaluated in the time-kill curve assay. When 1/2 MIC R-car was supplemented with 1/2 MIC S-car, a marked reduction was observed in the growth of MRSA following 4 h incubation, with complete growth inhibition following 16 h incubation (Fig. 2). The combination of 1/2 MIC R-car and 1/2 MIC GET caused rapid inhibition in a time-dependent manner after 4 h, with a complete inhibition of growth after 24 h (Fig. 3). The combination of 1/2 MIC S-car and 1/2 MIC GET markedly reduced the growth curve after 8 h and completely inhibited the growth of MRSA ATCC 33591 after 24 h (Fig. 4).

### Bacterial ultrastructure

**Figure 5:**
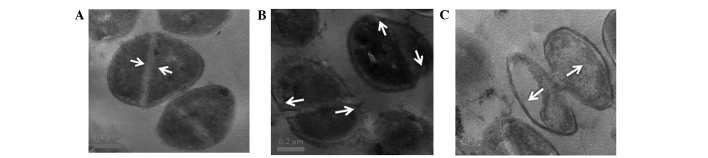
Transmission electron microscope images of MRSA subsequent to 24 h of R-car treatment. (A) Untreated control MRSA. These arrows indicate intact septa; (B) MRSA treated with 1/2 MIC R-car. These arrows indicate damage of the cell membrane caused by antimicrobial activity of R-car; (C) MRSA treated with 1 MIC R-car. These arrows show cell division, and cytoplasmic contents of the MRSA strains were out of the cell. MRSA, methicillin-resistant *Staphylococcus aureus*; MIC, minimal inhibitory concentration. Magnification, ×50,000.

Examination under an transmission electron microscope revealed cell lysis in R-car-treated MRSA cultures, which was the result of R-car-induced changes in cell division. Following 24 h exposure to 1/2 MIC R-car, MRSA cells were observed to have a damaged cytoplasmic membrane, while several ghosts of lysed cells were evident following 24 h treatment with 1 MIC R-car (Fig. 5).

## Discussion

Despite >50 years of investigation to identify novel antimicrobial agents against MDR strains, including MRSA, the emergence of resistant organisms has shown a global increase (15). Studies have suggested that medicinal plants and plant-derived compounds are necessary to overcome the problem of MDR infections, administered either alone or in combination with pre-existing antimicrobial therapies (16–18). In the present study, the anti-MRSA activities of R- and S-car in combination with GET were evaluated using an MIC assay. The MIC values of R- and S-car ranged between 500 and 1,000 μg/ml. Dual-agent therapy using different combinations of R-car, S-car and GET was examined using the checkerboard dilution assay. This combination strategy was used to enhance antibacterial potency relative to that of single drugs (19). The aminoglycoside antibiotic, GET, binds to the bacterial ribosome and disrupts its function (20).

In the checkerboard dilution experiment, synergistic anti-MRSA activity was observed against all six strains using any of the three dual-agent therapy combinations: R-car plus S-car, R-car plus GET or S-car plus GET. In the time-kill assay, the susceptibility of MRSA to the three treatment combinations was examined at 0, 4, 8, 16 and 24 h. Single-agent therapy did not induce cell death following 24 h incubation. By contrast, 1/2 MIC of any two agents in combination caused complete inhibition of bacterial growth following 24 h.

Uribe *et al* (21) reported that monoterpenoids, such as R- and S-car, exert an antimicrobial effect by interacting with the microbial membrane due to their inherent lipophilicity. In the present study, the synergistic effect elicited by the combination of R-and S-car suggested that carvones have a high affinity for the bacterial cell membrane and may influence structural or functional properties of the membrane (22). The synergy between either R- or S-car and GET indicated that R- and S-car have a major role in destroying the bacterial cell by increasing the permeability of the cell membrane, while GET is actively transported to the bacterial prokaryotic ribosome. These findings suggest a synergistic interaction between R- and S-car, as well as between carvone and GET. The mechanism of action of carvone against MRSA should be investigated in future studies.

Most antimicrobial agents cause membrane damage and cell lysis (23,24). In the present study, TEM revealed cytoplasmic disruption and separation of the cytoplasmic contents of MRSA following exposure to 1/2 and 1 MIC R-car. These changes in ultrastructure suggest that the MRSA cell membrane was damaged by R-car. In this study, the combination of R-car with S-car, and of either carvone enantiomer with GET exhibited significant anti-MRSA activity. These dual-agent combinations may reduce bacterial resistance to conventional antibiotics. The results of this study suggested that R- and S-car merit further investigation for the treatment of MRSA infection.

## Figures and Tables

**Table I:** MICs of R-car, S-car and GET against six strains of *Staphylococcus aureus*.

	MIC (μg/ml)		
	
*S. aureus*	R-car	S-car	GET
ATCC 25923	1000	1000	1.95
ATCC 33591	1000	1000	500
DPS-1	1000	1000	500
DPS-2	1000	1000	2000
DPS-3	1000	1000	1000
DPS-4	500	1000	500

MIC, minimal inhibitory concentration; R-car, R-carvone; S-car, S-carvone; GET, gentamicin.

**Table II:** Combination therapy of R-car plus S-car against methicillin-resistant *Staphylococcus aureus*.

		MIC (μg/ml)				
					
*S. aureus* strain	Agent	Alone	R-car + S-car	FIC	FICI	Outcome
ATCC 25923	R-car	1000	125	0.12	0.24	Synergy
	S-car	1000	125	0.12		
ATCC 33591	R-car	1000	62.5	0.06	0.12	Synergy
	S-car	1000	62.5	0.06		
DPS-1	R-car	1000	125	0.12	0.13	Synergy
	S-car	1000	15.6	0.01		
DPS-2	R-car	1000	125	0.12	0.24	Synergy
	S-car	1000	125	0.12		
DPS-3	R-car	1000	250	0.25	0.36	Synergy
	S-car	1000	125	0.12		
DPS-4	R-car	500	125	0.25	0.37	Synergy
	S-car	1000	125	0.12		

R-car, R-carvone; S-car, S-carvone; MIC, minimal inhibitory concentration; FIC, fractional inhibitory concentration; FICI, FIC index; DPS, *Staphylococcus aureus* strains from the Department of Plastic Surgery.

**Table III:** Combination therapy of R-car plus GET against methicillin-resistant *Staphylococcus aureus*.

		MIC (μg/ml)				
					
*S. aureus* strain	Agent	Alone	R-car + GET	FIC	FICI	Outcome
ATCC 25923	R-car	1000	31.25	0.03	0.09	Synergy
	GET	1.95	0.12	0.06		
ATCC 33591	R-car	1000	125	0.13	0.19	Synergy
	GET	500	31.25	0.06		
DPS-1	R-car	1000	125	0.13	0.38	Synergy
	GET	500	125	0.25		
DPS-2	R-car	1000	250	0.25	0.38	Synergy
	GET	2000	250	0.13		
DPS-3	R-car	1000	125	0.13	0.16	Synergy
	GET	1000	31.25	0.03		
DPS-4	R-car	500	125	0.25	0.28	Synergy
	GET	500	31.25	0.03		

R-car, R-carvone; GET, gentamicin; MIC, minimal inhibitory concentration; FIC, fractional inhibitory concentration; FICI, FIC index; DPS, *Staphylococcus aureus* strains from the Department of Plastic Surgery.

**Table IV:** Combination therapy of S-car plus GET against methicillin-resistant *Staphylococcus aureus*.

		MIC (μg/ml)				
					
*S. aureus* strain	Agent	Alone	S-car + GET	FIC	FICI	Outcome
ATCC 25923	S-car	1000	250	0.03	0.28	Synergy
	GET	1.95	0.48	0.25		
ATCC 33591	S-car	1000	125	0.13	0.19	Synergy
	GET	500	31.25	0.06		
DPS-1	S-car	1000	125	0.13	0.26	Synergy
	GET	500	62.5	0.13		
DPS-2	S-car	1000	125	0.13	0.18	Synergy
	GET	2000	125	0.06		
DPS-3	S-car	1000	250	0.25	0.31	Synergy
	GET	1000	62.5	0.06		
DPS-4	S-car	1000	250	0.25	0.31	Synergy
	GET	500	31.25	0.06		

S-car, S-carvone; GET, gentamicin; MIC, minimal inhibitory concentration; FIC, fractional inhibitory concentration; FICI, FIC index; DPS, *Staphylococcus aureus* strains from the Department of Plastic Surgery.

## References

[1] de Carvalho CCCR, da Fonseca MMR (2006). Carvone: Why and how should one bother to produce this terpene. Food Chem.

[2] Grodner B, Sitkiewicz D (2013). Enantiomers: a new problem in pharmacotherapy of depression?. Psychiatr Pol.

[3] Cho IJ, Lee CW, Lee MY, Kang MR, Yun J, Oh SJ, Han SB, Lee K, Park SK, Kim HM (2013). Differential anti-inflammatory and analgesic effects by enantiomers of zaltoprofen in rodents. Int Immunopharmacol.

[4] Krishnaiah YS, Nada A (2012). Enantioselective penetration enhancing effect of carvone on the in vitro transdermal permeation of nicorandil. Pharm Dev Technol.

[5] Lehmann K, Crombie A, Singer AC (2008). Reproducibility of a microbial river water community to self-organize upon perturbation with the natural chemical enantiomers, R- and S-carvone. FEMS Microbiol Ecol.

[6] Jäger W, Mayer M, Reznicek G, Buchbauer G (2001). Percutaneous absorption of the montoterperne carvone: implication of stereoselective metabolism on blood levels. J Pharm Pharmacol.

[7] Jevons MP (1961). ‘Celbenin’ - resistant Staphylococci. Br Med J.

[8] Tsubakishita S, Kuwahara-Arai K, Sasaki T, Hiramatsu K (2010). Origin and molecular evolution of the determinant of methicillin resistance in staphylococci. Antimicrob Agents Chemother.

[9] Ragle BE, Bubeck Wardenburg J (2009). Anti-alpha-hemolysin monoclonal antibodies mediate protection against *Staphylococcus aureus* pneumonia. Infect Immun.

[10] Gurieva TV, Bootsma MC, Bonten MJ (2012). Decolonization of patients and health care workers to control nosocomial spread of methicillin-resistant *Staphylococcus aureus*: a simulation study. BMC Infect Dis.

[11] Clinical and Laboratory Standards Institute (CLSI) (2006). Methods for dilution antimicrobial susceptibility tests for bacteria that grow aerobically: Approved standard.

[12] Chang SC, Chen YC, Luh KT, Hsieh WC (1995). In vitro activities of antimicrobial agents, alone and in combination, against *Acinetobacter baumannii* isolated from blood. Diagn Microbiol Infect Dis.

[13] Noble WC, Virani Z, Cree RG (1992). Co-transfer of vancomycin and other resistance genes from *Enterococcus faecalis* NCTC 12201 to *Staphylococcus aureus*. FEMS Microbiol Lett.

[14] Timurkaynak F, Can F, Azap OK, Demirbilek M, Arslan H, Karaman SO (2006). In vitro activities of non-traditional antimicrobials alone or in combination against multidrug-resistant strains of *Pseudomonas aeruginosa* and *Acinetobacter baumannii* isolated from intensive care units. Int J Antimicrob Agents.

[15] Moellering RC (2011). Discovering new antimicrobial agents. Int J Antimicrob Agents.

[16] Jung HJ, Lee DG (2008). Synergistic antibacterial effect between silybin and N,N′-dicyclohexylcarbodiimide in clinical *Pseudomonas aeruginosa* isolates. J Microbiol.

[17] Müller P, Alber DG, Turnbull L, Schlothauer RC, Carter DA, Whitchurch CB, Harry EJ (2013). Synergism between Medihoney and rifampicin against methicillin-resistant *Staphylococcus aureus* (MRSA). PLoS One.

[18] Celenza G, Segatore B, Setacci D, Bellio P, Brisdelli F, Piovano M, Garbarino JA, Nicoletti M, Perilli M, Amicosante G (2012). In vitro antimicrobial activity of pannarin alone and in combination with antibiotics against methicillin-resistant *Staphylococcus aureus* clinical isolates. Phytomedicine.

[19] Mun SH, Joung DK, Kim YS, Kang OH, Kim SB, Seo YS, Kim YC, Lee DS, Shin DW, Kweon KT, Kwon DY (2013). Synergistic antibacterial effect of curcumin against methicillin-resistant *Staphylococcus aureus*. Phytomedicine.

[20] Nester EW, Roberts CE, Pearsall NN, Anderson DG, Nester MT, Nester EW, Roberts CE, Nester MT (1998). Cell walls of gram-positive bacteria, cell walls of gram-negative bacteria. Microbiology: A Human Perspective.

[21] Uribe S, Ramirez J, Peña A (1985). Effects of beta-pinene on yeast membrane functions. J Bacteriol.

[22] Sikkema J, de Bont JA, Poolman B (1995). Mechanisms of membrane toxicity of hydrocarbons. Microbiol Rev.

[23] Denyer SP, Hugo WB (1991). Biocide-induced damage to the bacterial cytoplasmic membrane. Mechanisms of Action of Chemical Biocides: Their Study and Exploitation.

[24] Otto CC, Cunningham TM, Hansen MR, Haydel SE (2010). Effects of antibacterial mineral leachates on the cellular ultrastructure, morphology, and membrane integrity of *Escherichia coli* and methicillin-resistant *Staphylococcus aureus*. Ann Clin Microbiol Antimicrob.

